# The Science of the Human Body

**Published:** 1843-12

**Authors:** W. R. Handy

**Affiliations:** Professor of Anatomy and Physiology.


THE AMERICAN

### JOURNAL OF DENTAL SCIENCE.

Vol. IV.] DECEMBER, 1843. [No. 2.

### ARTICLE I.

### THE SCIENCE OF THE HUMAN BODY.

An Introductory Lecture, delivered before the Class of the Baltimore College of Dental Surgery, at the commencement of the Winter Course, Nov. 1843. By W. R. Handy, M. D., Professor of Anatomy and Physiology.

*Gentlemen of the Dental Class:—*

A distinguished author, in speaking of science, describes it as a "collection of facts, governed by laws," and these facts when looked at, we find beyond conception—in their number, variety, extent and relations. They cover no less a field than nature herself, comprehending her three great departments, viz. the mineral, the vegetable, and the animal kingdoms; and each of these departments is filled with a multitude of facts—hosts of which resemble each other so closely in their general form, size, common construction, and mode of operation, as to be considered identical in their very nature—and governed by the same general and common truths—which truths constitute so many *laws* belonging to the science, presiding over and embodying all such collection of similar facts. But the facts are not all alike, being in truth almost as various in kind, as they are multitudinous in number. Hence, every dissimilar collection of facts must have its own set of laws, and these laws must be referred to another and distinct science. Hence, it seems, that with the variety of facts, there must be a variety of laws, and a variety of sciences.

"There are in nature," says the celebrated Bichat, "two classes of beings, two classes of properties, and two classes of sciences: the beings are either organic, or inorganic; the properties vital, or non-vital—and the sciences physiological or physical.

It is not our intention here, even to enumerate the different sciences, much less to examine the various facts belonging to each; our object at this time being simply to direct attention to one science alone, viz. The science of the human body; a science which interests you, as its students, above every other science; forming, as it does, the sole basis on which alone a substantial reputation and prosperity, in the future practice of your profession, can securely rest; and the possession of which, each of you will doubtless, ardently and very properly aspire after.

It is, therefore, our earnest wish, gentlemen, to endeavor, if possible, to awaken in each of us, at this the outset of our course of studies, such an amount of proper feeling and degree of interest in the science of the human body, as will enable us to make a right and vigorous beginning, and pursue the same, step by step, faithfully and perseveringly to the end. To accomplish this, we propose presenting the claims of this science in some of its more attractive and cardinal features, as, viz.

1st. In its importance and utility.

2d. In its beauty, and,

3d. In its magnitude or extent.

The first point, viz. the great importance and utility of this science, all readily acknowledge, when its object is simply mentioned, viz. the preservation of health and life. An object as far surpassing that of every other object of science, as health does that of disease, or life that of death. An object, the possession of which, man has sacrificed every thing to attain;—wealth, business, friends—all these ties are and have been sundered;—time and distance consumed without measure, or hesitation;—oceans crossed—mountains scaled;—the world ransacked for its springs;—in a word, every thing considered as nothing in comparison of this object—health.

Well, the science of the human body is the only one, honored with the power of reaching this object; of infallibly guiding the diseased and worn out sufferer to it, and of finally rejoicing his heart by putting him in possession of it. This science thus preeminently exhibits itself over every other, in its importance and utility to mankind; and, by consequence of right, claims a proportionate superiority of all the dignity, respect, and gratitude, considered as belonging to any other science; in a word, it is the science of humanity, and to which, we shall see, as we pass along, all other sciences are more or less subservient and tributary.

The second feature, viz. the *beauty* of this science, is seen in the whole and every part of the human body. In the physical sciences we behold, for example, a watch, a specimen of the greatest ingenuity and skill; we are struck with admiration at the multitude and variety of its several parts, of their various form, size and direction; of their wonderful adaptation and harmony of action; and the no less astonishment, that the whole, notwithstanding being enclosed in so small a compass, and having such variety, should, nevertheless, all conspire, as by one common consent, to one great end, viz. the noting of time.

This example, all readily admit, is one of great beauty, both in design and skill—of design in the conception—of skill in the execution.

We behold a river having its origin in some distant and obscure spot, by a very small and insignificant beginning; we trace it on, step by step, as it descends the mountain and traverses the valley, and are struck with wonder and delight how this same body of water, which at first a mere point or speck, should become the mighty river before us, having thus swollen in majesty and strength in its onward march; being at one point the roaring cataract of the forest—at another the gentle visitor of the plain. This, every taste instinctively accords as an example both of great natural *beauty*, as well as utility in the natural world.

In the organic sciences, we behold as specimens in the vegetable department, the towering oak of the forest, and the beautiful lily of the valley. The former commencing by a small point or germ; in other words, is born—casts its tender roots in the earth, and sends its delicate trunk and branches in the air: the one descending deeper and deeper, the other higher and higher, till at last having reached its utmost development in size and strength, presents itself in all the *beauty* of its now full and matured proportions.

The lily, likewise, has its birth, growth and maturity, and expands and blooms in the richness of a dress, pronounced as surpassing in beauty all the splendors of Solomon; and in the animal department we behold the strength and ferocity of the lion, with the docility and gentleness of the lamb.

Yet, with all these specimens of beauty in the physical and organic sciences, and truly beautiful they are; with countless more equally so, what may we ask, are they all in comparison with the beauty of that which nature herself has pronounced her master-work, viz. the human body? for here we have a body possessing, in greater or less degree, the nature of every other body; and a science embracing the phenomena of both the physical and organic sciences, consequently combining the beauties of both; and hence, by necessity superior to either.

Have we admired the machinery of the watch, and its beautiful precision in noting time? Can this compare with the tenfold more complex machinery of the human body, and its still more beautiful precision in the preservation of health and life? Have we seen the beauty of the river, when stalking proudly in its course, and bearing on its bosom the riches of many soils, and fertilizing every where the land it visits? How much more have we to admire the circulation of the blood; a stream which moves onward without intermission from birth to death, and by a power superior to that of gravity; sending off endless ramifications in its course, which not only fertilizes the whole of the physical part of man, but further scatters its blessings, by so refreshing the human body, as to make it a living and fit temple for the exercise of those affections and faculties which peculiarly characterize its surpassing *beauty* over every other body.

We have also noticed, in the organic sciences, the beauty of the vegetable and the animal, in their birth, growth and full development in maturity. The human body has likewise the same physical charms of the plant, or the animal, as it goes through precisely the same stages of a birth, growth, and full development; but it has more, for in its several stages of infancy, childhood, youth, and manhood, there is added the additional beauty of the mind, which casts its intellectual and moral lustre over the physical frame of man, and making the whole and every part still more beautiful, whether seen in the eye, the countenance, or the gesture.

The following beautiful sentiment of the poet makes any further remarks unnecessary on this head; he exclaims: "How noble in reason! How infinite in faculties! In form and moving how express, and admirable! In action, how like an angel! In apprehension, how like a God! The *beauty* of the world! The paragon of animals!"

The third and last feature of the science of the human body we have to notice, is its *magnitude* or *extent*.

The human body, as already seen, being composed of both a physical and organic nature, its science is necessarily commensurate with, and embraces both the physical and organic sciences. But being likewise a mental body, it has, in addition, the vast field of the science of mind, comprising education and government.

Let us now briefly look at these several features in another and still more important aspect. We allude to the question of what constitutes the fundamental elements of the science of the human body? The answer to which will be found to embody a still more satisfactory explanation of the several features of this science, than what has just been advanced. These elements may be considered under three heads, viz. 1. A fixed and definite structure. 2. Fixed and definite functions, and, 3. Fixed and definite relations of structure and functions, with external or vital stimuli.

By the first element we include bone, muscle, blood-vessel, nerve, membrane, ligament, tendon, &c.; each of which has its own special and definite structure; embracing, likewise, the form, size, color, density and direction peculiar to each.

These several parts, variously combined among themselves, and with the fluids, compose the different organs of the body. Now, these organs, having as already stated, a fixed and definite structure, are endowed with a peculiar property or principle, of what, in an abstract view, may be considered over, and independent of the simple structure itself, and thereby gives to each particular structure, its own special value and interest. As, for instance, the *nerves* are endowed with the property of sensibility or feeling; take away this property and all our sensations and impressions would be gone; all the great highways of knowledge by the senses would be closed; and the mind of man, with his whole existence, a pure blank. The *muscles* are endowed with the property of contractility, or the power of shortening and lengthening themselves. Let us, for a moment, remove this property, and all the wheels of life immediately stop. The heart ceases to beat; the blood, by consequence, ceases to circulate, and the pulse is no longer felt. All motion in the limbs is gone; the hands cannot rise to gesture; the feet cannot move to walk; the eye cannot turn to see; neither can the mouth and larynx move to speak.

*Cartilage* has the property of elasticity; destroy this, and you at once destroy all that buoyancy, lightness, and ease of motion which characterise the graceful and natural step: take away this property, and the function of respiration, that most vital of all functions, will be most seriously injured, if not fatally destroyed.

*Bone* has the properties of solidity and strength; remove these, and the foundation of the human body is at once broken up; its form gone; its beauty fled; its strength vanished, and all the softer parts, having nothing to support them, must immediately tumble into a confused and shapeless mass.

But it is unnecessary to multiply further examples, showing that each particular structure has its own special property; as all we have in view, from this selection, is simply to set forth, if possible, in a still more conspicuous manner, the *importance, utility, beauty*, &c., constituting the prominent features of the science under discussion.

The second fundamental element named, is function. By this we mean, that every organ of the body has a distinct and special act or duty to perform, which act or duty is styled its function. The functions are various as, viz. those of circulation, respiration, sensation, secretion, &c.; which will come more particularly into notice at another time. This element is inseparably united with the first, in fact, cannot exist without it; the two go hand in hand, and both conjoined exhibit the science of the human body in two very distinct and opposite states, to wit: that of health and disease. These two states embrace the divisions of anatomy, physiology and pathology; and thus bring before us in a striking manner, another feature of this science, viz. its magnitude or extent.

The third and last element we propose to notice, is the relation or relations of structure and function. This being an important practical point, we shall examine it according to the several kinds of relations established by nature, as belonging to the different structures and functions. These may be reduced to three heads, viz. 1. The physical. 2. The organic, and 3. The mental relations.

The physical relations of the human body, are those it has with atmospheric air, water, aliment and heat. These four substances are termed vital stimuli; as they are indispensable, both to the existence and preservation of life; and the relations which these vital stimuli have with the different organs and functions, are so close and absolute, that we find nature has established them as so many fixed, positive, and unchangeable laws, for the government and well being of the body. Indeed, so unalterable and important are they in their character, that nature further backs their execution by bestowing the richest rewards for their observance, and inflicting the severest penalties for their violation. That such is the fact, a familiar example or two may be necessary. Every one knows and constantly feels the necessity of being able to breathe; and yet, if breathing be stopped but a few moments, in other words, if atmospheric air cannot be inhaled, life will certainly cease. Now, this is owing to the fixed and positive relation between the lungs and function of respiration, on the one hand, and the external vital stimulus atmospheric air on the other; and this relation, as just stated, is so fixed as to become an absolute law in every similar case. For, if we take any other air than atmospheric air and breathe it, we violate the law; because nature has made atmospheric air for the lungs, and will have no other substitute; and, if we persist, each will certainly suffer in proportion to the violation; that is, in the degree that the air taken into the lungs departs in its constituent elements from true, pure, atmospheric air. Who has not heard of well-diggers, instantly expiring by breathing a foul air, when reaching the bottom of the well, and so impure that a candle put into it, is immediately extinguished. How many thousands of the poor unfortunate miners of England have fallen victims to the choke-damp, a foul air; principally carbonic gas, and met with in their mining operations; we say, how many have thus lost their lives till the immortal discovery of the safety-lamp by Sir Humphrey Davy? But a still more familiar example is, the loss of life from breathing the fumes of charcoal, used as a substitute for the ordinary fire in the warming of bed rooms. Now, in all these instanccs we see the grossest violation of the law; for, in every case, the air breathed has been of the foulest and most impure kind, chiefly carbonic gas, a poison at once fatal to life, and as far as possible in its properties from atmospheric air. And what, the question occurs, do we witness as the penalty in all such cases of violation? The answer is familiar, that death, speedy death, is, in almost every instance, the dreadful consequence. It may be said, a person may breathe oxygen-gas, nitrous-oxyd-gas, and other airs, more or less impure, and all different from atmospheric air, and yet not die. These, however, form no exceptions to the law; for, it does not say, that all shall immediately die, but that all shall suffer in proportion to its violation. Now, the several airs, just mentioned, not being so poisonous as carbonic gas, just punish in the degree they are poisonous; so that we should naturally expect the symptoms and sufferings to vary in every case, precisely in the degree that the air is more or less impure, and differs from atmospheric air. On the other hand, let the atmospheric air be ever so pure, if we change the condition of the lungs and ribs, the law will still be violated. The lungs, for instance, are made to receive a certain quantity of air suited precisely to the wants of each particular case. If, now, from any cause whatever, they have their natural size or volume diminished, they become incapable of receiving this necessary quantity; which, though it may not immediately, will, in the end, as certainly destroy life, as too often seen in the case of consumption.

The fact that consumption exists, and is a fatal disease, and that too many of the fairer portion of our race fall its daily victims, all are ready to acknowledge; but how that too little air can produce it, may not be so clear; and, therefore, justify us in attempting a brief explanation.

When any compressing power is applied to the chest, such as the ordinary mechanical means in daily use for improving the beauty of the form, the ribs cannot rise and fall as nature designs they should; but, by being constrained and pressed in, they in turn press upon the natural cavity of the chest, and thus lessen the space in which the lungs are to play; hence, the lungs themselves are necessarily pressed upon, and they are, in turn, diminished in size. When in such condition, it is utterly impossible for the unhappy sufferer to breathe the quantity of air nature demands for healthy respiration; the consequence is, the two great functions, viz. respiration and circulation, become embarrassed. A short and hurried breathing announces the fact in the one case, while the prominent, blue and slow venous circulation as clearly shows it in the other. These symptoms report that the blood is not sufficiently changed in the lungs, from the fact of their being compressed to such an unnatural degree, that the required quantity of air cannot enter to meet the demands of the case; the necessary consequence being, the blood must leave the lungs unchanged, and thus go the round of the system, carrying its impurities to every part, and thus making every part to suffer. This state of things being continued day after day, the whole system becomes gradually undermined, the general health begins to fail, all the organs more or less to falter, and the whole body to waste. But still the patient may not be alarmed, nor friends suspect the real condition, when the truth is, consumption has already possessed the system, and tuberculous matter everywhere being deposited; but the fact fails to be discovered, or is not acknowledged, till too late to apply a remedy; not very often till hectic flush, night sweats, frightful cough, and growing emaciation, all too plainly show the real state of the case, and when death then is so near at hand that nothing can be done.

It is true, consumption may be hereditary, and arise from other causes; but this does not alter the fact, that a confined chest and crippled lungs, by preventing the proper quantity of air from being breathed—in other words, that the violation of nature's law of relation between the lungs on the one hand, and the air on the other—is not also a most frequent and unsuspected source of this most fatal disease?

The same may be urged for the rest of the physical relations, viz. water, aliment, and heat.

*Water* composes by far the greater portion of the fluids of the body, and greatly exceeds in weight that of the solids. Now nature is equally positive in this relation, as that of atmospheric air; her law is, that water is the natural fluid of the body, and that no other will answer; so that if man substitutes spirits, wine, or any other drinks, he violates the law, and must expect to suffer in proportion to the violation. It is unnecessary to multiply proof on this head, the bare mention of intemperance being all that is necessary to show the dreadful penalties which await the violators of this law.

And so with *aliment;* if we take poison instead of food, or food and fruits partly decomposed, and hence in a partial decay, the natural relation is destroyed, and the consequence is always injurious, and in many instances quickly fatal. And finally, as to *heat,* every one is aware that a certain amount is absolutely necessary for life; hence the necessity of the due observance of that law regulating the proper amount of clothing.

Now all these relations regard the body in a state of health, and belong to that department of the science we are examining denominated *hygiene.* But there yet remains another class of physical relations of a very different kind, and which regard the body in its opposite state, viz. disease; these are the remedies, constituting the *therapeutics* of the science; the simple mention of which, is all that we can here attempt.

We now pass to the second class of relations, viz. *the organic,* by which we mean the connection that subsists between the whole and every part of the body, usually termed sympathy; the body being a unit, and forming one complete whole, must necessarily have every organ to feel with and for every other organ. This being the case, when disease invades any portion of the system, all the rest being more or less closely related with the suffering organ, and having one common interest, must likewise expect to feel and suffer, and that generally in the proportion of the nearness of the connection, and the amount of influence each may have in the general economy. Hence disease of the stomach, liver, uterus, &c. &c. may affect the teeth, and disease of the teeth in turn affect either or all these organs; so that to understand any organ thoroughly, whether it be the teeth and their appendages, parts which are to claim your attention more particularly, it is absolutely necessary for us to study and become acquainted with every other organ—in other words, to study anatomy, and that kind of anatomy which can only be learned with the knife in the dissecting room.

Hence, gentlemen, it will be perceived that a proper knowledge of the dental profession is by no means limited, as too many are pleased to do, simply to the teeth alone; but, on the contrary, that these organs are proportionately commensurate in their relations and influence with all other organs; and hence, for the exercise of the proper skill in the detecting and treating their diseases, or letting them alone altogether, which is often preferable, belongs peculiarly to the dentist. And hence, we say, to exercise a conscientious judgment in your profession, not only a knowledge of every other organ besides the teeth is necessary, but also no small share of the science of medicine in general.

The last class of relations belonging to the science of the human body is, viz. *the mental:*—that the mind and body are closely related, and reciprocally influence each other, no one pretends to question; the fact of sudden and disastrous news immediately destroying the appetite, and of fever producing delirium, are sufficient evidences of the close union of the two.

The mind also, has relations with the external world, and through the medium of the bodily senses; this is a fixed law of nature, and remains such, notwithstanding all that mesmerism says to the contrary; nature declares by this law that all our knowledge must come through the senses—mesmerism says no; nature affirms the law positive and absolute as all her other laws, and consequently appends penalties for its violation; while she, on the other hand, bestows the richest rewards for obedience. Knowledge is the priceless treasure given for observance, while ignorance is the heavy penalty for violation.

Lastly, the mind has a further and very extensive set of relations, viz. with all other minds. This relation communes with the dead as well as the living, it regards the past, and looks forward to the future. By this relation the science of the human body is greatly extended, and owes its highest and proudest elevation: for, from this communion of mind with mind, the action of each on the other, and the perpetual conflict thus carried on, do we owe all the high and inestimable privileges of civilization, as science, government, the arts, &c. &c. By it, our knowledge of the external world is made pure, precise, and practical. By it, our natures tend to higher and still higher stages of continually progressive elevation and refinement. And, finally, out of it and to it do all other sciences owe their origin and bring their treasures.

This hasty view of these several relations has, in another light, brought before us, as it were in a single glance, all the prominent features that have been mentioned as belonging to the science of the *human body*, viz. its *importance* and *utility*, *beauty* and *magnitude.*

And in conclusion, gentlemen, we trust these points have been so exhibited and illustrated, as not only to show the inherent value and dignity of your science, but what has been the main burden of all we have been trying to say, viz. to awaken, at the outset, in each of you, a proper and lively interest for its honor and usefulness, and to impress the fact that it is only by constant and persevering study on your parts, that you can, in the first place, hope to obtain a reputable acquirement of this science; and then, in the second place, maintain inviolate its honor, and be a useful and vigilant member, in promoting its onward advancement.

*Baltimore, Nov.* 1843.

### ARTICLE II.

*Extract from a Letter to the Baltimore Editor.* By DR. J.C. MCCABE.

